# Serum Leptin and Ghrelin Levels in Women with Polycystic Ovary Syndrome: Correlation with Anthropometric, Metabolic, and Endocrine Parameters

**Published:** 2012-06-19

**Authors:** Shiva Houjeghani, Bahram Pourghassem Gargari, Laya Farzadi

**Affiliations:** 1 Students’ Research Committee, Faculty of Health and Nutrition, Tabriz University of Medical Sciences, Tabriz, Iran; 2 Nutritional Research Center, Department of Biochemistry and Diet Therapy, Faculty of Health and Nutrition, Tabriz University of Medical Sciences, Tabriz, Iran; 3 Department of Obstetrics and Gynecology, Faculty of Medicine, Tabriz University of Medical Sciences, Tabriz, Iran

**Keywords:** Polycystic Ovary Syndrome, Leptin, Ghrelin, Insulin

## Abstract

**Background:**

Polycystic ovary syndrome (PCOS) patients are more prone to abnormal production of some regulatory peptides. In these patients, studies on the serum levels of leptin and ghrelin are controversial. This study aims to investigate serum levels of leptin and ghrelin and their correlation with metabolic and endocrine indices in PCOS.

**Materials and Methods:**

This case-control study was conducted on 60 women; 30 with PCOS and 30 healthy women whose age and body mass index (BMI) were matched and who were referred to Alzahra Hospital, Tabriz, Iran. Serum levels of leptin, ghrelin, insulin, luteinizing hormone (LH), follicle stimulating hormone (FSH), sex hormone-binding globulin (SHBG), and testosterone were measured. The homeostasis model assessment of insulin resistance (HOMA-IR) was calculated. Descriptive statistics and correlations were performed using SPSS 12.0 for Windows.

**Results:**

In PCOS women, serum levels of leptin, insulin, HOMA-IR, testosterone, LH, and LH/FSH were significantly higher, while SHBG was lower than in healthy women. Ghrelin and FSH were similar in both groups. Serum levels of leptin correlated with BMI (r=0.85, p<0.001), waist to hip ratio (WHR) (r=0.55, p<0.01), insulin levels (r=0.85, p<0.001) and HOMA-IR (r=0.67, p<0.01), while ghrelin levels had an inverse association with testosterone (r=-0.32, p=0.04).

**Conclusion:**

The results showed increased leptin levels while ghrelin remained unchanged in PCOS patients. In PCOS patients, leptin positively correlated with BMI, WHR, insulin, and insulin resistance, while ghrelin was only associated with serum testosterone levels.

## Introduction

Polycystic ovary syndrome (PCOS) affects approximately 6–10% of women of reproductive age and is characterized by ovarian dysfunction, hirsutism, hyperandrogenism, insulin resistance, and obesity (1). The etiology of PCOS is multifactorial, including both genetic and environmental issues. Although hyperandrogenism, ovarian dysfunction, abnormalities in the hypothalamic-pituitary axis, and excess insulin activity are known to be responsible for pathogenesis of the syndrome, the exact etiology has yet to be discovered (1, 2).

Obesity is a very common clinical feature in women affected by PCOS. More than 50-60% of PCOS women are obese (3). Adiposity is observed in PCOS patients and plays an important role in their metabolic phenotype through the production of various adipocyte-derived cytokines and proteins known as adipokiens (4). Central obesity has a close relationship with the altered secretion of some adipocytokines like leptin. Production of adipocytokines affects insulin sensitivity and is a predictor of metabolic syndrome (5).

Leptin, the product of the ob (obese) gene, is a single-chain 16 kDa protein consisting of 146 amino acid residues. Leptin is produced mainly in adipose tissue and is involved in the regulation of energy homeostasis, reproduction, insulin action, and lipid metabolism. The relationship between leptin and reproductive function is complex and not completely understood (6).

Leptin is a key hormone in energy homeostasis and neuroendocrine function and has a permissive role in the pathogenesis of reproductive dysfunction (7). Recent studies suggest that some hormones may mediate some of the adverse effects of obesity on ovarian function in PCOS (8-10). Studies on the leptin in PCOS have conflicting results; some show increased levels of leptin (11), while others show no difference in leptin in PCOS compared to healthy subjects (12-14).

Ghrelin, an endogenous ligand for the growth hormone secretagogue receptor, is synthesized principally in the stomach. It stimulates food intake and transduces signals to hypothalamic regulatory nuclei that control energy homeostasis and are linked to the control of key aspects of reproduction function. The peptide consists of 28 amino acids (15). Studies on the ghrelin levels in PCOS are conflicting. Orio et al. (16) have shown no difference in ghrelin levels among PCOS and healthy controls. However Wasko et al. (17) noted high ghrelin levels, while Mitkov et al. (10) and Kamal et al. (18) showed low levels of ghrelin in PCOS patients compared to the control group. Glintborg et al. (14) have found that ghrelin levels decreased in hirsute PCOS patients.

There is evidence of leptin and ghrelin operating as endocrine-paracrine mediators, establishing a link between energy homeostasis and reproduction (19). The major site of these novel mediators of the appetite is the central nervous system (CNS), especially the hypothalamus and pituitary, where they affect gonadotropin-releasing hormone (GnRH), pulsatility, follicle stimulating hormone (FSH), and luteinizing hormone (LH) production and secretion (20, 21).

Contradictory results in studies investigating serum leptin and ghrelin encouraged us to carry out the current research. We further assessed the association between LH, FSH, testosterone, sex hormone-binding globulin (SHBG), body mass index (BMI), waist-tohip ratio (WHR), and insulin resistance, with the two above-mentioned hormones.

## Materials and Methods

The present case-control study was conducted on 30 PCOS patients and 30 healthy patients matched for age, BMI, and WHR that were referred to Alzahra Hospital in Tabriz, Iran. Sampling lasted from November 2008 to February 2009. The study protocol was approved by the Ethics Committee of the Tabriz University of Medical Sciences.

After being informed of the purpose and procedures of the study, all subjects signed an informed consent form. The diagnosis of PCOS was made by a gynecologist using Rotterdam criteria, which includes clinical and/or biochemical signs of hyperandrogenism (increased serum total testosterone or free androgen index), oligomenorrhoea (six or fewer menses per year) or amenorrhoea (no menses in the last six months), and polycystic ovaries (by ultrasonographic examination) (22).

Medical history, physical and pelvic examination, and complete blood tests were used to determine the healthy status of women in the control group. Exclusion criteria for all subjects included pregnancy, hypothyroidism, hyperprolactinemia, Cushing’s syndrome, congenital adrenal hyperplasia, current or previous (within the last three months) use of oral contraceptives, glucocorticoids, antiandrogens, ovulation induction agents, antidiabetic and anti-obesity drugs, or other hormonal drugs. None of the patients were affected by any neoplastic, metabolic, or other concurrent medical illness. Weight and height were measured to calculate the BMI. Body height was measured to the nearest 0.1 cm with the subject standing without shoes. Body weight in light indoor clothing was measured to the nearest 0.1 kg. The BMI was calculated using the standard formula of weight (kg)/height (m^2^). Waist and hip circumferences (at the level of the hipbone anterior superior iliac spines) were also measured in the standing position to calculate the WHR.

The analyses were carried out during the early follicular phase (days 3-5) in women who had menstrual cycles, and in any phase of the cycle in PCOS patients. Basal blood samples were obtained to evaluate serum leptin, ghrelin, LH, FSH, total testosterone, SHBG, fasting insulin, and glucose levels. All blood samples for each subject were assayed in duplicate and immediately centrifuged. The serum was stored at -80˚C until analysis.

In each woman, the estimate of insulin resistance by homeostasis model assessment of insulin resistance (HOMA-IR) was calculated with the following formula: fasting serum insulin (mU/l) × fasting plasma glucose (mg/dl)/405 (23).

All blood samples were obtained between 08:00 am and 09:00 am after an overnight fast. The serum leptin level was measured using a Human Leptin ELISA Kit (BioVendor GmbH. Im Neuenheimer Feld 583. D-69120; Heidelberg, Germany), which had an intra-assay and inter-assay coefficient of variation, 4.2-7.6%, and 4.4-6.7%, respectively and sensitivity of 0.2 ng/ml. In all subjects, plasma immunoreactive ghrelin levels were measured using a commercially available RIA that uses 125I-labeled bioactive ghrelin as a tracer and a rabbit polyclonal antibody raised against full-length octanoylated human ghrelin (Phoenix Pharmaceuticals Inc., Belmont CA, USA), which recognizes both acylated and des-acylated ghrelin. Levels of serum LH and FSH were determined by direct immunoenzymatic method (DiaMetra S.r.l; Bartolomei, Z.I Paciana, Folingo (PG), Italy). The intraassay CVs of the assays used were: 7.9% (LH) and 9.4% (FSH). The inter-assay CVs of the assays used were: 9.0% (LH) and 11.8% (FSH).

The measurement of serum SHBG was performed using an enzyme-linked immunosorbent assay (ELISA) kit (IBL Immuno-Biological Laboratories; Flughafenstrasse 52A, D-22335, Hamburg, Germany) with an intra-assay CVs of 3% and inter-assay CVs of 8.7%. Total testosterone levels were determined using a commercially available ELISA kit (Monobind Inc., 100 North Pointe Drive, Lake Forest, CA 92630, USA) which had an intra-assay CV of 5.2% and an inter-assay CV of 6 %.

All parameters studied or calculated showed normal distributions, which were confirmed by the one sample Kolmogorov-Smirnoff test. Results were expressed as mean ± standard deviation (SD). Comparisons between the two groups were made using an independent samples t test. Pearson correlation analyses were performed to define correlations between parameters. p<0.05 was regarded as statistically significant. All analyses were run using the SPSS (version 12.0, SPSS, Chicago, IL).

## Results

**Fig 1:**
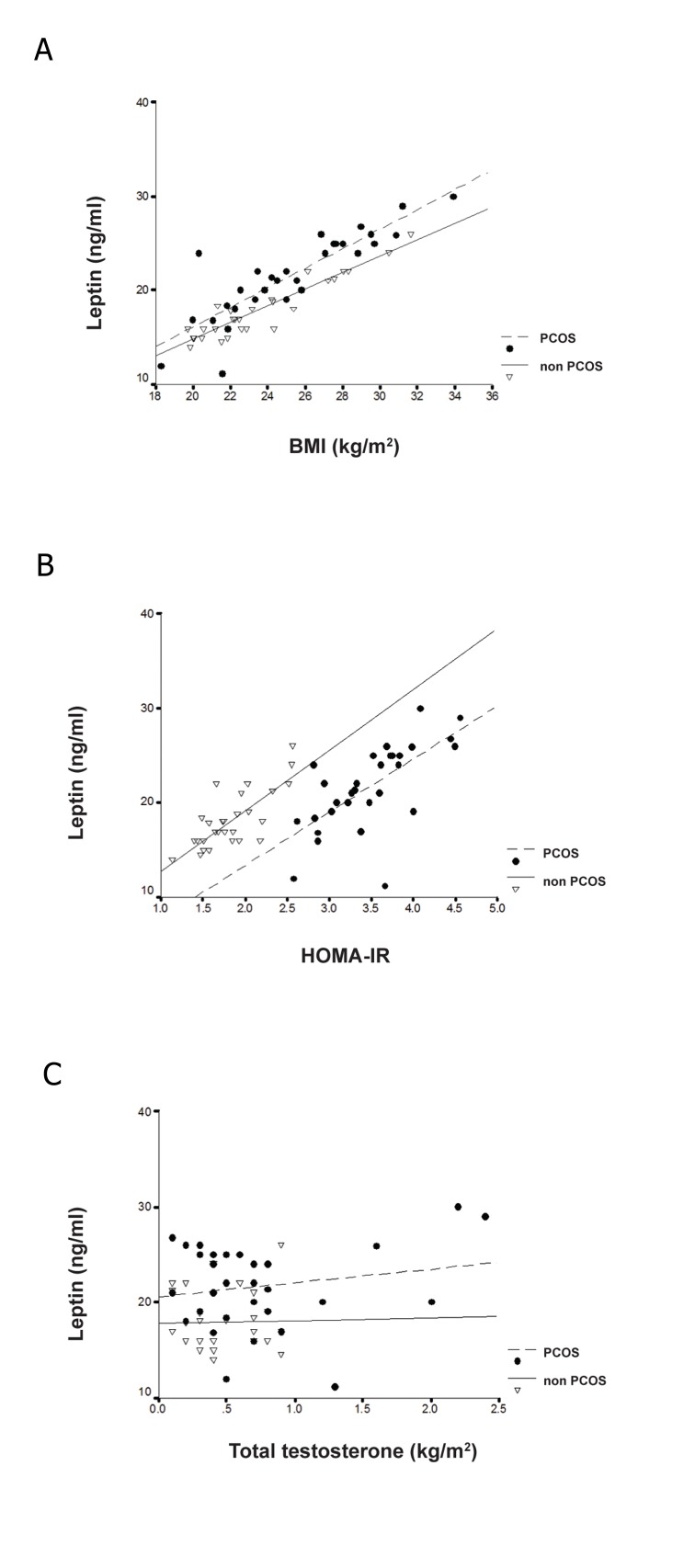
(A) Correlation between serum leptin levels and BMI (PCOS; r=0.85, p<0.001,Controls; r=0.93, p<0.01). (B) HOMA- IR (PCOS; r=0.67, p<0.01, Controls; r=0.77, p<0.01). (C) Total testosterone (PCOS; r=0.19, p>0.05, Controls; r= 0.02, p>0.05).

There were 30 women were in the PCOS group and 30 in the control group. The anthropometric and laboratory data of the groups are presented in table 1. Figure 1 shows mean serum levels of ghrelin. Data confirmed that the subjects in the healthy control group matched subjects in the PCOS group in terms of age, BMI, and WHR (Table 1).

**Table 1:** Anthropometric, metabolic, and hormonal characteristics of patients and controls


	PCOS (n=30)	Control (n=30)

**Age (Y)**	25.83± 4.00	26.06± 4.44
**Weight (Kg)**	64.40± 1 0.46	62.4± 8.82
**Height (cm)**	160.1 ± 6.01	162.4± 6.53
**BMI (Kg/m^2^)**	25.00± 3.61	23.68± 3.07
**Waist (cm)**	81.01± 8.98	79.53± 6.4
**Hip (cm)**	101.00 ± 6.37	99.13± 6.35
**WHR**	0.80± 0.56	0.80± 0.60
**Leptin (ng/ml)**	21.68± 4.49**	17.96± 3.00
**Ghrelin (pmol/l)**	210.33 ± 58.5	216.00 ± 80.84
**Insulin (mu/l)**	14.91± 1.78**	7.90± 1.16
**Glucose (mg/dl)**	92.6± 8.3	94.4± 8.6
**HOMA- IR**	3.47± 0.54**	1.81± 0.36
**Total testosterone (ng/ml)**	0.75± 0.60*	0.45± 0.26
**SHBG (ng/ml)**	31.81 ± 14.29*	52.34 ± 23.41
**LH (mIU/ml)**	12.50± 2.33**	4.86± 2.12
**FSH (mIU/ml)**	6.03 ± 1.64	5.74 ± 1.10


Data presented as means ± SD. BMI; body mass index, FSH; follicle-stimulating hormone, HOMA-IR; homeosta-sis model assessment of insulin resistance, LH; luteinizing hormone, PCOS; polycystic ovary syndrome, SHBG; sex hormone binding globulin and WHR; waist-to-hip ratio. *p<0.05, **p<0.001.

In the PCOS group, serum levels of leptin, insulin, HOMA-IR, testosterone, LH, and LH/ FSH were significantly higher than in the control group. SHBG concentration was found to be lower in the PCOS group. As for ghrelin and FSH, no significant difference was detected in either group.

**Fig 2:**
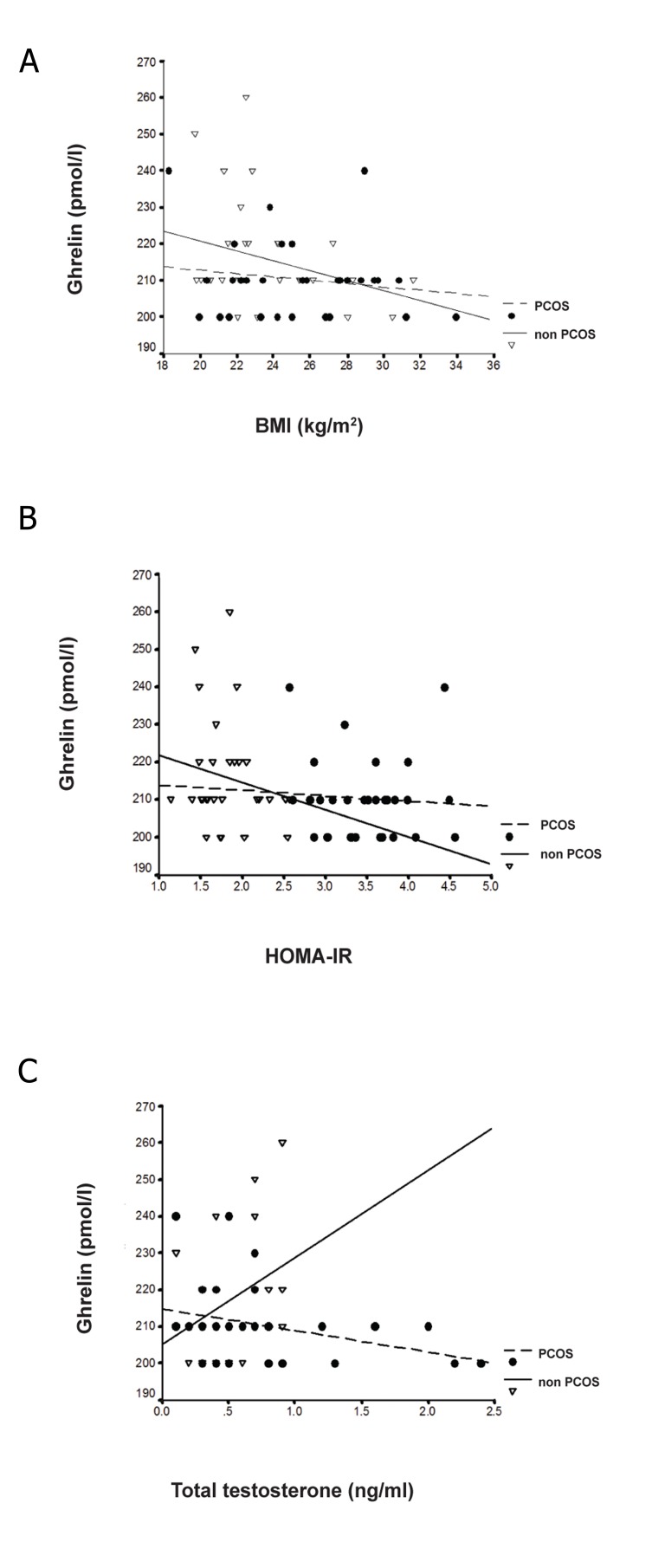
(A) Correlation between serum Ghrelin levels and BMI (PCOS; r=-0.04, p>0.05, Controls; r=-0.22, p>0.05). (B) HOMA-IR (PCOS; r=-0.12, p>0.05, Controls; r=-0.14, p>0.05). (C) Total testosterone (PCOS; r=-0.32, p<0.05, Controls; r=-0.42, p>0.05).

Bivariate correlations (Table 2 and Fig 2) revealed that serum levels of leptin in PCOS women significantly correlated with BMI (r=0.85, p=0.001), WHR (r=0.55, p=0.01), insulin levels (r=0.85, p=0.001) and HOMA-IR (r=0.67, p=0.01).

**Table 2:** Pearson correlation tests of clinical, metabolic, and hormonal parameters with leptin and ghrelin in the study groups


	PCOS (n=30)		Control (n=30)	

	Leptin	Ghrelin	Leptin	Ghrelin

**Weight (Kg)**	0.74**	-0.24	0.80**	-0.17
**BMI (Kg/m^2^)**	0.85***	-0.04	0.93**	-0.22
**Waist (cm)**	0.80**	-0.23	0.59*	-0.11
**WHR**	0.55**	-0.19	0.10	-0.10
**Insulin (mu/l)**	0.85***	-0.09	0.86**	-0.26
**HOMA-IR**	0.67**	-0.12	0.77**	-0.14
**LH (mIU/ml)**	0.22	0.16	0.35	-0.02
**FSH (mIU/ml)**	- 0.11	0.13	0.29	-0.31
**LH/FSH**	-0.17	0.22	0.16	0.26
**Total testosterone (ng/ml)**	0.19	-0.32*	0.02	-0.42*
**SHBG (ng/ml)**	-0.11	0.15	-0.07	0.67**


FSH; Follicle-stimulating hormone, HOMA-IR; Homeostasis model assessment of insulin resistance, LH; Luteinizing hormone, SHBG; Sex hormone binding globulin and WHR; Waist-to-hip ratio. * p<0.05, **p<0.01, ***p<0.001.

There was no significant correlation of ghrelin to weight, BMI, WHR, insulin, HOMA-IR, FSH, and LH in both groups. A significant inverse association was found between ghrelin and testosterone levels in both the PCOS (r=-0.32, p=0.04) and the control group (r=-0.42, p=0.02) (Fig 2). Testosterone levels tended to correlate positively with BMI (r=0.32, p=0.04) and waist circumference (r=0.40, p=0.03) in PCOS women.

Insulin levels significantly correlated with BMI and waist circumference in both the PCOS group and the control group. BMI (r=0.78, p=0.001) and waist circumference were also positively associated with HOMA-IR (r=0.71, p=0.001).

## Discussion

Findings from current research show that women with PCOS had higher levels of insulin, HOMAIR, testosterone, LH, and LH/FSH and lower concentrations of SHBG.

Higher leptin levels may have a role in the pathophysiology of PCOS. Leptin has a dual effect on reproduction. The positive effect of leptin is its role as a trigger of puberty on hypothalamicpituitary axis by stimulating estrogen secretion. The negative impact of leptin, in conditions like hyperleptinemia is the inhibition of the ovarian response to gonadotrophin stimulation (24).

Studies of leptin levels in PCOS women have yielded conflicting results. Similar to the findings of Mitkov et al. (10) and Pehlivanov et al. (11) we showed an increased level of leptin in PCOS patients. Other authors have failed to show any difference among PCOS and healthy women (12-14). It can be said that differences in age, anthropometric indices of groups, or the severity of the disease can account for these divergent results.

A correlation between serum leptin and BMI has been shown in PCOS women (25, 26). Our findings confirm the result of previous studies, showing a significant correlation between BMI and waist circumference with leptin. This further supports the importance of abdominal fat mass in the secretion of leptin.

SHBG is a glycoprotein produced in the liver acting as a carrier for different sexual steroid hormones. It shows a higher affinity for testosterone (27). The concentration of SHBG is stimulated by cortisol, estrogens, and growth hormone and decreased by androgens, insulin and prolactin (28). Lower SHBG levels caused by hyperinsulinemia may be responsible for the increased bioavailability of sex hormones in target tissues (29). This may in turn lead to the development of abdominal obesity.

Insulin resistance and subsequent hyperinsulinemia are found in 50-70% of PCOS patients. High insulin levels are associated with hyperandrogenism and anovulation (6, 7, 30). It has been proven that insulin is able to stimulate ovarian steroidogenesis (30), and increase ovary LH receptors and the sensitivity of pituitary gonadotropes to GnRH action (31).

The steady-state basal serum glucose and insulin concentrations are determined by their interaction in a feedback loop. A computer model is used to predict the homeostatic concentrations that result from varying degrees of insulin resistance and β-cell deficiency. Comparison of a patient’s fasting levels with the model’s predictions provides a quantitative assessment of the contributions of deficient β-cell function and insulin resistance to the fasting hyperglycemia (homeostasis model assessment; HOMA) (23). In our study we applied the HOMA index to evaluate the status of insulin resistance in the two study groups.

In the present study the PCOS group showed higher insulin levels, HOMA-IR, elevated testosterone, and decreased SHBG levels than the healthy controls.

Peripheral (hepatic and skeletal muscle) insulin sensitivity and pancreatic β-cell function is improved via leptin action in these sites (32). Insulin stimulates both leptin biosynthesis and secretion from adipose tissue, creating an endocrine adipoinsular feedback loop called the "adipo-insular axis" (33). On the other hand, in clinical interventional studies, postprandial and short term hyperinsulinemia using euglycemic-hyperinsulinemia clamp studies have been unable to show an increase in leptin secretion (34).

Current research has revealed that in the PCOS and control group leptin was positively correlated with insulin levels and HOMA-IR. There is evidence of leptin ability in stimulating GnRH from the hypothalamus and LH/FSH release from the pituitary (20, 21). Recombinant human leptin treatment in patients with hypothalamic amenorrhea has been reported to increase the mean LH levels and LH pulse frequency within two weeks of receiving treatment (35).

Studies in which 24 hour LH pulses were observed in PCOS patients (36) showed an inverse relationship between leptin and 24 hour mean LH levels. The loss of bi-hormonal synchrony between leptin and LH release was reported by Veldhuis et al. (37). A positive association between leptin and serum LH levels was demonstrated by Atamer et al. (9). In agreement with Mendonça et al. (38), we did not observe a relation between leptin and LH in either the PCOS or control group.

Studies investigating the association of leptin with LH that measured 24 hours LH and leptin pulses provide a more precise, sensitive, and objective index of alterations in bi-hormonal linkage than studies using a single measurement of serum concentration of these two hormones, as used in our study. However, the involvement of leptin in modulating LH and FSH via its pulsatile secretory characteristics has yet to be elucidated in either healthy or PCOS subjects.

It has been shown that most obese individuals need higher doses of gonadotropins for ovary hyper-stimulation, despite comparable absorption of gonadotropins from subcutaneous tissue (39). There are also reports of leptin having an inhibitory effect on the synergic action of FSH and insulin- like growth factor I on granulosa cell estradiol production (40).

However, similar to Rouru et al. (41), our research did not show a relationship between leptin and serum FSH levels. The lack of a significant relationship may be explained in this way: leptin reduces FSH function not solely by reducing FSH serum levels; some other mechanisms such as a decrease of FSH receptors in granulosa cells might be implicated. Effects of leptin on FSH level are more noticeable when it is exogenously administered and *in vitro* studies may also demonstrate a more clear perspective of a probable association.

In hypogonadal men, testosterone supplementation has been shown to normalize elevated leptin concentrations without any changes in body fat or BMI (42). Significant correlations between leptin and serum testosterone have not been found in all studies. However, an inverse association has been reported in both untreated and testosterone-treated hypogonadal men (43). Similar to Haffner et al., we did not detect a correlation between leptin and testosterone (44). We believe leptin may modulate testosterone levels in PCOS through its effect on insulin concentrations. Since hyper-insulinemia is associated with high leptin concentrations (45), and the role of insulin in regulating a key step of androgen formation (regulation of P450c17 enzyme) (46), it is probable that leptin affects androgen levels via its impact on insulin secretion instead of a direct alteration of serum testosterone.

Lower levels of SHBG probably mirror a higher testosterone to estrogen ratio (47). Some studies have shown that SHBG levels may have an effect on altered leptin levels by weight loss which may be due to improvements in leptin sensitivity in these subjects (48). Leptin concentration might indirectly have a relationship with SHBG levels in PCOS subjects in our study. PCOS patients with higher leptin levels compared to healthy women have lower SHBG concentrations. Our findings support the findings from other studies that found no correlation between leptin and SHBG levels in women with hirsutism (49) and subjects with PCOS (41).

In our study, ghrelin levels did not show a significant difference between the two groups. In the literature, obese PCOS compared to obese healthy subjects have been found to have lower ghrelin levels, but when lean and obese PCOS groups were taken as a whole and compared to BMImatched controls, ghrelin levels were found to be similar between both groups (35). Orio et al. (16) and Daghestani et al. (50) showed similar results. In studies conducted by Mitkov et al. (10), Glintborg et al. (14), and Kamal et al. (18), serum ghrelin concentration was reported to be lower in the PCOS group than in healthy controls. Despite these results, Wasko et al. (17) have reported elevated levels of plasma ghrelin in PCOS patients compared to healthy controls. This discrepancy of results may be explained by confounding factors, such as body weight, fat mass, age, hormonal status, and severity of disease.

Similar to the findings of our study, Schofl et al. (51) showed that ghrelin level did not correlate with BMI. On the other hand, Daghestani et al. showed a significant inverse relationship between ghrelin and BMI in both PCOS and healthy subjects. It must be said that WHR was significantly different between the two groups in the Daghestani et al. (50) study.

It has been shown that ghrelin administration to healthy humans at pharmacological doses reduces insulin secretion (52), and conversely, insulin administration at high doses is capable of reducing ghrelin secretion (53). Our results have shown that, despite differences in circulating insulin levels, there was not a significant correlation between insulin levels and HOMA-IR with fasting ghrelin concentrations. The reason why our results have not shown this relation might be that insulin or ghrelin are able to affect each other when they are administered at pharmacological doses.

In humans the specific effects of ghrelin on LH secretion have not been indicated. It is feasible that more comprehensive analyses involving precise assessment of LH pulsatility after ghrelin administration might reveal a subtle regulatory role of ghrelin in the control of gonadotropin secretion in humans. We have found no correlation between ghrelin levels and serum LH, FSH, or LH/FSH ratio in PCOS and control groups. These finding do not support the idea that ghrelin might alter gonadotropin levels.

The ghrelin receptor is found not only in the CNS but also in the ovarian tissues, suggesting a possible reproductive function (54). Moreover, the capability of ghrelin to alter stimulated testosterone secretion *in vitro* has been documented (55). Using bivariate correlations we found a significant inverse association between total testosterone and ghrelin in both study groups. In previous studies there were reports of no significant association (51) and an inverse association (56) between serum levels of ghrelin and testosterone.

In the current research, we further examined the association of testosterone with BMI and waist circumference. As the BMI and waist circumference increased, the serum testosterone levels showed a significant elevation. These findings have demonstrated that the increase of body weight and fat tissue is associated with abnormalities in sex steroid balance.

Some limitations of the present study were the relatively low sample size, narrow range of BMI, and the measuring of leptin and ghrelin only in a fasting state. Matching subjects for age and anthropometric indices have been considered as strengths of our study. However, to reach a better understanding of PCOS pathophysiology, more studies are warranted in which PCOS patients are grouped based on their BMI, insulin and androgen levels, presence of clinical features of hyperandrogenism, and severity of polycystic ovaries. Ghrelin and leptin or other hormones should be precisely measured in both fasting and postprandial states in relation to endocrine parameters.

## Conclusion

The findings of this study have suggested that indices of adiposity (BMI and WHR) are responsible for elevated leptin, insulin resistance, and testosterone levels in PCOS patients. The role of leptin and ghrelin in the pathogenesis of PCOS may occur by ways other than the simple concentration of these hormones in circulation, particularly as leptin inserts its endocrine effects mostly through modulating insulin levels.
